# Bradykinesia in dystonic hand tremor: kinematic analysis and clinical rating

**DOI:** 10.3389/fnhum.2024.1395827

**Published:** 2024-06-13

**Authors:** Peter Matejicka, Slavomir Kajan, Jozef Goga, Igor Straka, Marek Balaz, Simon Janovic, Michal Minar, Peter Valkovic, Michal Hajduk, Zuzana Kosutzka

**Affiliations:** ^1^2nd Department of Neurology, Faculty of Medicine, Comenius University, Bratislava, Slovakia; ^2^Institute of Robotics and Cybernetics, Faculty of Electrical Engineering and Informatics, Slovak University of Technology in Bratislava, Bratislava, Slovakia; ^3^1st Department of Neurology, St. Anne’s University Hospital, Masaryk University, Brno, Czechia; ^4^Central European Institute of Technology (CEITEC), Brno, Czechia; ^5^Institute of Normal and Pathological Physiology, Center of Experimental Medicine, Slovak Academy of Sciences (SAS), Bratislava, Slovakia; ^6^Department of Psychology, Faculty of Arts, Comenius University, Bratislava, Slovakia; ^7^Centre for Psychiatric Disorders Research, Science Park, Comenius University in Bratislava, Bratislava, Slovakia; ^8^Department of Psychiatry, Faculty of Medicine, Comenius University, Bratislava, Slovakia

**Keywords:** dystonic tremor, bradykinesia, finger tapping, kinematic analysis, blinded clinical rating

## Abstract

**Introduction:**

Bradykinesia is an essential diagnostic criterion for Parkinson’s disease (PD) but is frequently observed in many non-parkinsonian movement disorders, complicating differential diagnosis, particularly in disorders featuring tremors. The presence of bradykinetic features in the subset of dystonic tremors (DT), either “pure” dystonic tremors or tremors associated with dystonia, remains currently unexplored. The aim of the current study was to evaluate upper limb bradykinesia in DT patients, comparing them with healthy controls (HC) and patients with PD by observing repetitive finger tapping (FT).

**Methods:**

The protocol consisted of two main parts. Initially, the kinematic recording of repetitive FT was performed using optical hand tracking system (Leap Motion Controller). The values of amplitude, amplitude decrement, frequency, frequency decrement, speed, acceleration and number of halts of FT were calculated. Subsequently, three independent movement disorder specialists from different movement disorders centres, blinded to the diagnosis, rated the presence of FT bradykinesia based on video recordings.

**Results:**

Thirty-six subjects participated in the study (12 DT, 12 HC and 12 early-stage PD). Kinematic analysis revealed no significant difference in the selected parameters of FT bradykinesia between DT patients and HC. In comparisons between DT and PD patients, PD patients exhibited bigger amplitude decrement and slower FT performance. In the blinded clinical assessment, bradykinesia was rated, on average, as being present in 41.6% of DT patients, 27.7% of HC, and 91.7% of PD patients. While overall inter-rater agreement was moderate, weak agreement was noted within the DT group.

**Discussion:**

Clinical ratings indicated signs of bradykinesia in almost half of DT patients. The objective kinematic analysis confirmed comparable parameters between DT and HC individuals, with more pronounced abnormalities in PD across various kinematic parameters. Interpretation of bradykinesia signs in tremor patients with DT should be approached cautiously and objective motion analysis might complement the diagnostic process and serve as a decision support system in the choice of clinical entities.

## 1 Introduction

Dystonia frequently presents with concomitant tremor, and when such tremor manifests in regions affected by dystonia, it is categorized as dystonic tremor (DT). Clinically, DT is a jerky and irregular tremor usually absent at rest, sometimes with the presence of geste antagoniste, which may attenuate the tremor and the null point with the absence of tremulous activity. The diagnosis of DT is based on clinical assessment/evaluation, with no specific laboratory tests. DT is often misdiagnosed as essential tremor (ET) because subtle dystonic posturing may be missed/overlooked by clinicians (Jain et al., 2006; Murgai and Jog, 2020). DT typically occurs either simultaneously with or after the onset of dystonia (Deuschl, 2003). However, there are cases where DT can occur years before the emergence of dystonia, leading to diagnostic uncertainties. On the other hand, dystonic activity may influence the execution of repetitive movements like finger tapping (FT) and hand movements that may be mistaken for bradykinesia. In the last decade, the presence of bradykinesia in non-parkinsonian disorders has also emerged (Paparella et al., 2021) and the need for more accurate recognition of bradykinesia led to a very recent redefinition proposal (Bologna et al., 2023). Identification of movement slowness points clinicians towards a differential diagnosis of parkinsonian disorders. Additionally, due to the subjective rating of bradykinesia, assessment is also burdened with high intra-rater and inter-rater variability and that consequently leads to low overall reliability (Arceneaux et al., 1997; de Deus Fonticoba et al., 2019). Nevertheless, the crucial point in correct recognition of limb bradykinesia is its definition per se, which is still not firmly established (Schilder et al., 2017). For example, for the diagnosis of PD, bradykinesia is defined as slowness of movement and decrement in amplitude or speed (or progressive hesitations/halts) as movements are continued (Postuma et al., 2015). The sensitive discriminative feature in favour of PD is the sequence effect or decrement that is defined as the progressive slowing of sequential movements (amplitude and velocity decrement with repetitive and continuing movements) (Benecke et al., 1987). The sequence effect is missing in ET patients, but these patients are usually slower than healthy controls (Bologna et al., 2020). Amplitude decrement might also be missing in patients with the progressive supranuclear palsy (PSP) (Ling et al., 2012), but other studies did not find any differences in features based on blinded video ratings between PSP and PD (Marsili et al., 2023). Moreover, the sequence effect of bradykinesia can be missed by the naked eye. This has led to the development of objective motion capture systems that measure the specific attributes of finger tapping such as velocity, amplitude, frequency, sequence effect and many others with clinically meaningful outcomes, helping in differential diagnosis of movement disorders (Hasan et al., 2017).

The aim of the current study is two-fold: (1) to assess the kinematic features of finger tapping by an optical hand tracking system in patients with a clinical and neurophysiological diagnosis of DT; (2) to assess FT bradykinesia by means of three independent experienced movement disorder specialists.

## 2 Participants and methods

All participants were recruited from the Centre for Movement Disorders at the 2nd Department of Neurology, University Hospital Bratislava.

The inclusion criteria were:

• Consecutive patients with DT in the hands classified according to the consensus of Bhatia et al. (2018) given an axis I classification thus, according to its phenomenology and clinical characteristics−action tremor of the upper limbs accompanied by at least one of the following clinical or electrophysiological signs: dystonic posture, motor overflow, antagonist muscles co-contraction, mirror dystonia, geste antagoniste, null point, and/or subcortical myoclonus (Bhatia et al., 2018). The surface polymyography used as a supporting factor for the diagnosis of DT was performed using a Neurosoft^®^ Neuro-MEP-8 EMG machine. We followed a protocol created by Apartis et al. (Apartis, 2013) for electrophysiological verification of the dystonic features of the tremor. The whole protocol can be found in the Supplementary material. Patients were absent of tremors in any other body part than in the upper limbs.
• Consecutive patients with PD, fulfilling the Postuma criteria (Postuma et al., 2015) in the early stage of the disease (Hoehn and Yahr stage 1–2) with tremor and bradykinesia in at least one hand.
• Healthy controls (HC) of similar age.

All patients underwent DaT-SPECT examination with positive findings corresponding with a supposed diagnosis of PD and negative findings in DT patients. Healthy controls did not undergo the DaT-SPECT examination. An MRI scan of the brain was performed on all subjects, including healthy controls, to exclude any structural lesions. Evaluation of cognitive functions was performed using the Montreal Cognitive Assessment (MoCA) (Nasreddine et al., 2005).

Exclusion criteria in all participants were diseases other than PD and DT (neurologic or orthopaedic) that could affect finger tapping performance, and MoCA tests less than or equal to 25 points that could be suggestive of cognitive deficit. Each subject signed an informed consent form before the study. The study was conducted in accordance with the Declaration of Helsinki and the study was approved by the ethical committee of University Hospital Bratislava Nr. 2/2020.

Clinical examination of all patients and HC consisted of taking a recent medical history (all previous medical history was known before meeting the inclusion criteria) and a general neurological examination. For indicative exclusion of some mild cognitive impairment or dementia, the MoCA test was performed. In PD patients, part III (motor examination) of MDS-UPDRS was evaluated. The patients with DT were rated with the Fahn-Tolosa-Marin Tremor Rating Scale (Fahn et al., 1988). In patients with DT, all medication for tremor was stopped at least 24 h before data acquisition. In patients with PD, dopaminergic treatment was stopped 24 hours before in the case of dopamine agonists and 12 hours before in the case of levodopa before data acquisition; patients were thus examined in the OFF medication stage.

### 2.1 Finger tapping recording by optical hand tracking system

Clinical assessment was followed by kinematic analysis using an optical sensor - Leap Motion Controller (LMC). The patient was sitting at the table and the LMC was resting flat on the table in front of the patient. The computer screen connected with the LMC sensor was hidden from the patient’s view and the most suitable position for the hand during measurement is approximately in the middle in front of the sensor and at a height of 30 cm. We instructed the patient to tap the index finger on the thumb as quickly and as big as possible for 10 sec as the standard protocol. This time interval is sufficient to provide evidence of a typical decrement in PD patients and does not necessarily induce movement fatigue. The same procedure was repeated with the contralateral hand. We chose 10 s periods for each hand due to the relatively quick fatigue of forearm muscles, especially in DT patients. The position of the LMC varied with the examined hand. The subjects were asked to sequentially perform the same task alternately three times for both upper limbs. All trials were also recorded from the front position on a video camera. This helped us assess the accuracy of the measurement if there were problems with the LMC recordings (bad position of the hand, overlapping of fingers and others) and was also used for blinded video rating.

#### 2.1.1 Data processing and extraction of kinematic variables

Raw data from the LMC sensor in the form of two grayscale stereo images for the right and left cameras captured in the near-infrared light spectrum (850 nm) were streamed via USB and subsequently processed by the sensors’ hand tracking software. The LMC measures physical quantities in the following units: distance (mm), time (μs), speed (mm.s^–1^), and angle (rad). By applying advanced algorithms for the compensation of ambient lighting and background objects, the image data are analysed and used to create a 3D representation of the hand model within the range of the sensor provided by the LMC software. Our real-time bradykinesia application collected image data and data from a 3D hand model using the LMC software. All measured data between 0 and 10 sec of the recording were analysed. During the FT test, the amplitude was calculated as the Euclidean distance between the fingertips of the index finger and the thumb (maximal vs. minimal separation). The measured data were not sampled by the same sampling period, so we resampled them with a 5 ms period using linear interpolation. In addition to amplitude, other variables such as speed and acceleration of finger movement were also calculated. The overview of analysed parameters can be seen in Table 1. Validation of the LMC sensor measurement was done with an accelerometer and a gyroscope measurement system. In the first validation an accelerometer was used, which was fixed to the distal phalanx of the index finger and which measured the acceleration of the movement. The signal was detected with a single-axis acceleration transducer and recorded on a Neurosoft^®^ Neuro-MEP-8 EMG machine. In this validation, the measured frequency of finger tapping was compared if the errors were less than 0.5%. Secondly, the validation employed a measurement system using gyroscopes, which was created as described by Djurić-Jovičić. In this validation, the shape agreement of the measured amplitude was compared if the agreement of the amplitudes was high, similar to the paper (Djurić-Jovičić et al., 2017).

**TABLE 1:** Description of the calculated kinematic parameters.

Kinematic parameter	Description
Amplitude	Computed from the peak values of a periodic signal−peak value is the maximum positive deviation of the waveform from its zero reference level (global minimum value)
Amplitude decrement	The difference between the amplitudes at time of first maximum and last sample obtained by linear regression from the amplitude signal in the given period. The same method was used in the publication by Bologna et al., 2020
Frequency	Computed as f = 1/T where f is the frequency in hertz, and T is the maximum period of a signal in seconds
Frequency decrement	The difference between the frequencies at time of first maximum and last sample obtained by linear regression from the frequency signal in the given period
Speed	Mean finger tapping speed (the mean of the first derivative of the Savitzky-Golay filtered amplitude signal, all samples being taken into account in the calculation)
Acceleration	Mean finger tapping acceleration (the mean of the second derivative of the Savitzky-Golay filtered amplitude signal, all samples being taken into account in the calculation)
Halts	Number of skips of a period (pauses) within a signal. A period is defined by a decrease in amplitude by 10% and tapping speed by 40% relative to neighbouring peaks

### 2.2 Blinded video rating of finger tapping by movement disorder specialists

Anonymised FT videos recorded during the second trial of kinematic analysis using LMC trial (of both hands) were presented to movement disorder specialists with more than 10 years’ experience in the field. Videos were shown in randomised order using the Excel tool for randomisation. Three raters were blinded to the experimental protocol and the diagnoses of the patients including the healthy controls. They had to rate bradykinesia (presence or absence) as defined in the latest clinical criteria for PD (Postuma et al., 2015), also stating the reason for bradykinesia (slowness of movement AND decrement in amplitude or speed or progressive hesitations/halts). The presence of bradykinesia in every subject was defined when present at least in one or both hands. The video was focused only on the hand part. Other parts of the body, if they were unwittingly recorded, were blurred.

**FIGURE 1:**
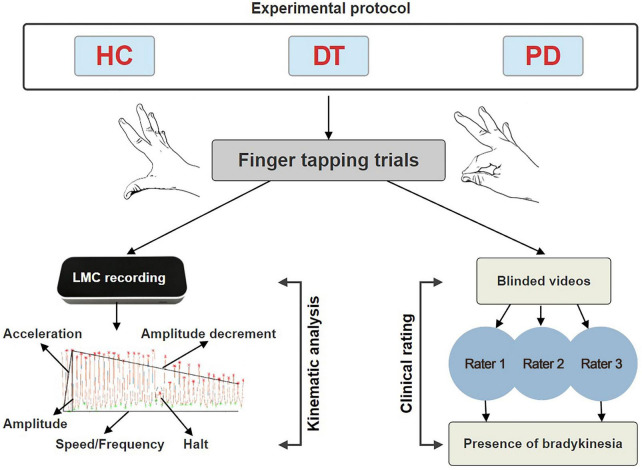
Experimental protocol consisting of kinematic analysis and blinded clinical rating of finger tapping. HC, healthy controls; DT, patients with dystonic tremor; PD, patients with Parkinson’s disease; LMC, Leap motion controller.

The outline of study protocol is depicted in Figure 1.

### 2.3 Statistical analyses

We compared groups using a linear mixed model analysis (Brown, 2021). Kinematic analysis parameters were entered as dependent variables, diagnosis (HC vs. DT vs. PD) as a fixed effect, and subject ID as a random effect. We used this approach because we had several measures from each patient and linear-mixed models might improve statistical power in the smaller samples. Estimated marginal means were compared using contrast analysis. Covariates were not included in the model, because this might have impacted the ability to estimate such a complex model on a small sample. A probability value (*p*) of *p* < 0.05 was considered significant for all the analyses.

The presence of bradykinesia was reported as percentages from the total count of participants in each group. Agreement analysis was performed to assess inter-rater reliability. Degree of agreement was measured with Fleiss’ Kappa (*κ*).

Differences in kinematic parameters between the left and right hands were compared using a paired sample *t*-test (Ross and Willson, 2017). Due to the small sample size, we did not reflect handedness nor disease side severity in further analysis. All statistical analysis was performed using JASP (version 0.16.4.0).

## 3 Results

A total of 36 subjects met the inclusion and exclusion criteria and were analysed. The demographic and clinical characteristics are presented in Table 2.

**TABLE 2:** Demographic and clinical characteristics of the study population.

	Healthy controls (*n* = 12)	Patients with PD (*n* = 12)	Patients with DT (*n* = 12)
Age (years; mean ± SD)	60 ± 7.37	56.2 ± 5.95	65.36 ± 8.29
Gender (W:M)	7:5	5:7	8:4
Handedness (R:L)	9:3	8:4	8:4
Symptom Predominance (R:L)	NA	3:9	4:8
Disease duration (years; mean ± SD)	NA	2.2 ± 0.79	5.68 ± 3.52
MDS-UPDRS part III−OFF medication (mean ± SD)	NA	30.5 ± 10.95	NA
Hoehn and Yahr stage−OFF medication (median, IQR)	NA	2 ± 1	NA
FMT Scale part A/B (mean ± SD)	NA	NA	11.75 ± 8.5/13.82 ± 8.4
MoCA (mean ± SD)	28.1 ± 1.78	26.2 ± 1.48	26.64 ± 1.91

PD, Parkinson’s disease; DT, dystonic tremor; SD, standard deviation; IQR, interquartile range; W:M, women:men; R:L, right:left; MDS-UPDRS, The Movement Disorder Society-Sponsored Revision of the Unified Parkinson’s Disease Rating Scale; FTM, Fahn-Tolosa-Marin Tremor Rating Scale; MoCA, Montreal Cognitive Assessment; NA, not assessed.

### 3.1 Kinematic analysis

**FIGURE 2:**
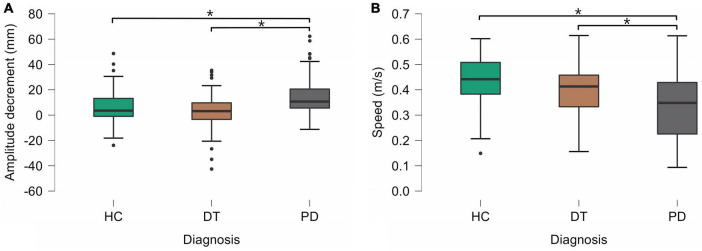
Statistically significant differences between groups in amplitude decrement **(A)** and speed **(B)**. HC, healthy controls; DT, patients with dystonic tremor; PD, patients with Parkinson’s disease.

Using linear mixed models we estimated marginal means (EMM) with standard errors (SE). The effect of diagnosis was significant in amplitude decrement (*F* = 9.058, *p* < 0.001), speed (*F* = 5.231, *p* = 0.012), and acceleration (*F* = 3.587, *p* = 0.041). Kinematic analysis revealed no significant difference in the selected parameters of FT bradykinesia between DT patients and HC. In comparisons between DT and PD patients, PD patients exhibited bigger amplitude decrement [DT–EMM = 2.7 (2.14) mm, PD–EMM = 15.93 (2.4) mm] and slower FT performance [DT–EMM = 0.4 (0.02) m/s, PD–EMM = 0.33 (0.03) m/s]. Acceleration of FT was significantly slower only when comparing HC and PD [HC–EMM = 22.6 (1.53) m/s^2^, PD– EMM = 16.36 (1.75) m/s^2^]. The group effect in the amplitude parameter was close to the threshold but did not reach statistical significance (p = 0.097) therefore the result of post hoc analysis (HC had bigger amplitude of FT than patients with PD) should be taken with caution. All post-hoc group comparisons (based on the contrast) are presented in Table 3 as well. The graphical depiction of group differences is depicted in Figure 2.

**TABLE 3:** Estimated marginal means of kinematic parameters with inferential statistics.

	HC mean (SE)	DT mean (SE)	PD mean (SE)	*F* test	Group differences
Amplitude (mm)	68.09 (3.24)	61.44 (3.35)	57.28 (3.74)	*F* = 2.525 *p* = 0.097	HC > PD
Amplitude decrement (mm)	6.16 (2.1)	2.7 (2.14)	15.93 (2.4)	*F* = 9.058, *p* < 0.001*	HC < PD, DT < PD
Frequency (Hz)	3.67 (0.22)	3.65 (0.23)	3.22 (0.25)	*F* = 1.127, *p* = 0.338	
Frequency decrement (Hz)	0.22 (0.09)	0.42 (0.09)	0.37 (0.1)	*F* = 1.374, *p* = 0.270	
Speed (m/s)	0.44 (0.02)	0.4 (0.02)	0.33 (0.03)	*F* = 5.231, *p* = 0.012*	HC > PD, DT > PD
Acceleration (m/s^2^)	22.6 (1.53)	20.05 (1.59)	16.36 (1.75)	*F* = 3.587, *p* = 0.041*	HC > PD
Halts	1.06 (0.28)	1 (0.3)	1.52 (0.32)	*F* = 0.830, *p* = 0.446	

HC, healthy control; PD, Parkinson’s disease; DT, dystonic tremor; SE, standard error; mm, millimeter, Hz, Hertz; m, meter; s, second. Significance is marked with bold and asterisk.

### 3.2 Bradykinesia rating by a movement disorder specialist

Rater 1 (M.B.) reported bradykinesia based on finger tapping in 7 out of 12 DT patients (58.3%), in 4 out of 12 (33.3%) of the HC, and in all 12 patients with PD (100%).

Rater 2 (I.S.) reported bradykinesia based on finger tapping in 4 out of 12 DT patients (33.3%), in 3 out of 12 (25%) of the HC, and in 10 out of 12 PD patients (83.3%).

Rater 3 (M.M.) reported bradykinesia based on finger tapping in 4 out of 12 DT patients (33.3%), in 3 out of 12 (25%) of the HC, and in 11 out of 12 PD patients (91.7%).

Bradykinesia was found on average among raters in 41.6% of DT patients, 27.7% of HC, and 91.7% of PD patients.

Agreement analysis showed average moderate inter-rater agreement in evaluating the presence or absence of bradykinesia in the combined sample with κ = 0.553 (95% CI 0.365–0.742).

In the DT group, the overall agreement between raters was weak [κ = 0.086 (95% CI 0.241– 0.412)] and in the HC strong [κ = 0.723 (95% CI 0.396–1.000)]. In the PD group, the agreement was fair with κ = 0.273 (95% CI −0.054−0.599). Individual ratings of the presence/absence of bradykinesia are displayed in Supplementary Table 1.

## 4 Discussion

In the current study, we assessed FT bradykinesia in the group of patients with clinical and neurophysiological diagnosis of DT from two points of view−its presence, rated by movement disorder specialists, and the severity, using an optical sensor. To our knowledge, this is the first study to assess signs of bradykinesia in a group of patients with DT. Based on blinded clinical rating, bradykinesia as defined in the latest MDS consensus statement was reported in almost half of the DT patients in our cohort. Still, kinematic parameters did not differ between HC and DT patients.

One possible explanation is that our kinematic analysis was not sensitive enough to depict subtle changes in the selected parameters. On the other hand, it was sensitive enough to detect the differences between DT and PD patients. When comparing the kinematic analysis between DT and PD patients, the DT patients had smaller amplitude decrement and faster FT performance, suggesting a similar performance to HC. This observation does not preclude the manifestation of bradykinesia in DT patients, rather, it elucidates a quantitatively milder degree of severity. The speed and decrement can often be missed by less trained healthcare professionals or, on the other hand, overrated by movement disorder specialists, thus favouring the use of validated sensor-based assessment as decision support. Kinematic analysis might meaningfully complement clinical assessment in diagnostically uncertain cases with good sensitivity, especially when diagnosing PD (Garcia-Agundez and Eickhoff, 2021) and assessing bradykinesia in other tremor-related aetiologies (Butt et al., 2018; Lee et al., 2019).

From a pathophysiological point of view, DT seems closer to nontremulous dystonia than ET (Panyakaew et al., 2022). Based on a recent review article by Paparella et al., bradykinesia is frequently observed in dystonia involving basal ganglia, primary sensorimotor cortex and cerebellum in the pathological mechanism (Paparella et al., 2023). Mild parkinsonian signs are an additional manifestation of dystonia arising mainly from the already mentioned basal ganglia dysfunction (Haggstrom et al., 2017). Still, the prevalence of bradykinesia in limb dystonia has also not been fully elucidated.

Our kinematic analysis did not show any differences in the total number of halts between HC and clinical groups even though they are anchored in MDS diagnostic criteria of bradykinesia. Halts are likely not PD-specific and can be also present in other diseases and healthy populations. This statement can be confirmed by other studies reporting movement interruptions in other parkinsonian and non-parkinsonian disorders. Moreover, there is a lack of meaningful correlation between halts and bradykinesia severity (Yu et al., 2023), and the number of halts does not change with dopaminergic medication (Thijssen et al., 2022).

Interestingly, movement disorder specialists found amplitude or speed decrement in 67% of the DT patients. The accurate depiction of amplitude decrement undeniably requires an experienced clinical eye and might be overlooked or sometimes overrated in routine clinical practice. In either case, the observed decrement in patients with dystonia could be attributed to aberrant sensorimotor integration, dystonic activity induced by movement that may mimic a genuine decrement, or straightforward peripheral fatigue (Tinaz et al., 2016). This contrasts with ET and PSP, where the decrement is missing, even though the studies used objective kinematic analysis and not assessment by movement disorder specialists (Ling et al., 2012; Bologna et al., 2020).

The false identification of bradykinesia could be affected by interruptions in movement resulting from dystonic activity and tremors, potentially impeding the accurate assessment of FT bradykinesia ratings. The overflow of dystonic activity may give rise to spasms and incoordination when performing FT tasks that might mimic bradykinesia (Hallett, 2000). High rater disagreement in the case of DT patients could also point to incorrect execution of the FT movement due to tremulous activity or dystonic overflow causing abnormal postures. Additionally, the inter-rater variability of FT is common when assessing PD patients showing a high percentage of incongruent ratings (Bennett et al., 1997; Goetz and Stebbins, 2004). The clinicians in our study rated bradykinesia in almost 28% of HC. This is comparable with a recent study by Williams et al. that reported the presence of bradykinesia in 24% of HC (Williams et al., 2023). Bradykinesia might be a part of normal ageing. Age-related changes in the human brain’s functioning fundamentally affect the motor system, causing increased reaction time, low ability to control movements, and difficulties in learning new motor skills (Frolov et al., 2020). The performance decrements observed in older adults are also attributed to inferred strategic preferences for accuracy over speed (Saling and Phillips, 2008).

The occurrence of FT bradykinesia in clinical ratings may result from the diverse range of diagnoses that could have been present in our cohort, despite our meticulous patient selection process involving thorough clinical examinations, neurophysiological assessments, and DaT-SPECT imaging.

Most probably patients with scans without evidence of dopaminergic deficits (SWEDDs) could be incorporated into our DT group. Indeed, SWEDDs with parkinsonian signs may present as adult onset DT patients including bradykinetic features (Schneider et al., 2007). However, we did not screen for other supporting factors favouring SWEDDs, e.g., orthostatic hypotension, cardiovascular and thermoregulatory dysfunction. Neither did we trial response to dopaminergic treatment.

Essential tremor is another clinical entity that could have been included in our DT cohort, but clear dystonic activity was confirmed by the neurophysiology. Based on the tremor classification by Bhatia et al. (2018), DT patients could also meet the criteria of essential tremor-plus. However, ET-plus is still a controversial topic with unclear conclusions. Dystonia with parkinsonism is, of course, a known entity of different aetiologies, but it is not entirely typical when dealing with patients of the age of our sample (Morales-Briceno et al., 2022). It is unlikely that there would be so many genetically determined forms of dystonia-parkinsonism syndromes in our DT group. Unfortunately, none of the patients in our cohort had genetic testing. Finally, individuals with corticobasal syndrome may exhibit asymmetric DT with negative DaT-SPECT results, particularly in the initial phases (Constantinides et al., 2019). However, it is noteworthy that all subjects underwent comprehensive structural MRI scans, enabling the detection of asymmetric cortical atrophy.

### 4.1 Limitations

The limitations of our study must be considered. First of all, we acknowledge the small sample size in each group, but the main limitation was the number of DT patients who would meet the strict inclusion criteria used to carefully select homogeneous patients for each group. The signs of bradykiensia in all groups were assessed based solely on upper limb FT performance; other bradykinetic features (hypomimia, lower limb and gait assessment) were not rated. Nevertheless, in clinical practice FT is considered to be the most sensitive and feasible way to identify bradykinetic features.

Disease stage/severity (in PD patients according to MDS-UPDRS and in DT patients according to the FTM scale), as well as the age composition of patients in the groups, may also have affected the results. However, although patients with DT had longer disease duration compared to PD patients, the relatively slow progression of DT means it is unlikely that this factor would have had an impact on the results. Although all PD and DT patients underwent DaT-SPECT, the healthy controls did not undergo this examination because of the ionizing radiation from radioactive substances and CT scanning; thus, patients with dopaminergic denervation could have been included in the study.

We should also mention the direct limitations of the LMC device, such as view angle, reliability in terms of precision of finger position and error rates over 20% (Gamboa et al., 2020). Patient compliance during LMC recordings may play a role in parameter acquisition, such as the hand moving out of the camera’s view, flexion of other fingers except for the index finger and thumb, and supination or pronation of the hand during measurement. It seems that hand tremor did not influence the recording but our study did not focus on tremor, only on bradykinesia.

## 5 Conclusion

In conclusion, individuals with DT exhibit no discernible differences in kinematic parameters compared to HC; however, in clinical evaluations, these patients may manifest authentic bradykinesia, including the presence of decrement. Bradykinesia, thus, represents a non-specific symptom observed in healthy populations and non-parkinsonian disorders. The results of our study further confirm that kinematic analysis might add valuable information about selected parameters of bradykinesia in non-parkinsonian patients and increase diagnostic accuracy.

## Data availability statement

The original contributions presented in the study are included in the article/Supplementary material, further inquiries can be directed to the corresponding author.

## Ethics statement

The studies involving humans were approved by the Ethics Committee University Hospital Bratislava−Kramare, Limbova 5, 83305 Bratislava, Slovakia. The studies were conducted in accordance with the local legislation and institutional requirements. The participants provided their written informed consent to participate in this study.

## Author contributions

PM: Writing−original draft, Writing−review and editing, Conceptualization, Data curation, Formal analysis, Methodology, Project administration, Supervision, Visualization. SK: Writing−original draft, Writing−review and editing, Software, Validation, Visualization. JG: Software, Validation, Visualization, Writing−review and editing, Writing−original draft. IS: Writing−review and editing, Conceptualization, Data curation, Supervision. MB: Data curation, Writing−review and editing. SJ: Data curation, Writing−review and editing. MM: Data curation, Writing−review and editing, Supervision, Validation. PV: Supervision, Writing−review and editing. MH: Writing−review and editing, Methodology. ZK: Writing−review and editing, Methodology, Writing−original draft, Conceptualization, Data curation, Formal analysis, Project administration, Supervision, Funding acquisition.
