# Supplementary material for: Bradykinesia in dystonic hand tremor: kinematic analysis and clinical rating
Source: Front Hum Neurosci. 2024 Jun 13;18:1395827. doi: 10.3389/fnhum.2024.1395827 (PMC11208697; doi:10.3389/fnhum.2024.1395827)
Supplement: Supplementary file 1 [file Data_Sheet_1.docx]

**Supplementary file**

Supplementary Table 1. Blinded ratings of bradykinesia in different groups (0 - absent, 1 - present, PD - patients with PD, R- right-hand side, L - left-hand side, BRADY - bradykinesia)

|  | **RATER 1** | | | | | | | **RATER 2** | | | | | | | **RATER 3** | | | | | | |
| --- | --- | --- | --- | --- | --- | --- | --- | --- | --- | --- | --- | --- | --- | --- | --- | --- | --- | --- | --- | --- | --- |
|  | **slowness** | | **decrement** | | **halts** | | **BRADY** | **slowness** | | **decrement** | | **halts** | | **BRADY** | **slowness** | | **decrement** | | **halts** | | **BRADY** |
|  | **R** | **L** | **R** | **L** | **R** | **L** |  | **R** | **L** | **R** | **L** | **R** | **L** |  | **R** | **L** | **R** | **L** | **R** | **L** |  |
| **PD1** | 1 | 1 | 1 | 1 | 0 | 0 | **YES** | 0 | 0 | 1 | 0 | 1 | 1 | **NO** | 0 | 0 | 1 | 0 | 1 | 1 | **NO** |
| **PD2** | 1 | 1 | 1 | 1 | 1 | 1 | **YES** | 0 | 1 | 1 | 1 | 1 | 1 | **YES** | 0 | 1 | 1 | 1 | 1 | 1 | **YES** |
| **PD3** | 1 | 1 | 0 | 1 | 0 | 1 | **YES** | 1 | 1 | 0 | 1 | 0 | 1 | **YES** | 1 | 1 | 0 | 1 | 0 | 1 | **YES** |
| **PD4** | 1 | 1 | 1 | 1 | 1 | 1 | **YES** | 1 | 1 | 1 | 1 | 1 | 1 | **YES** | 1 | 1 | 1 | 1 | 1 | 1 | **YES** |
| **PD5** | 1 | 1 | 0 | 1 | 0 | 0 | **YES** | 0 | 0 | 1 | 1 | 1 | 1 | **NO** | 1 | 1 | 1 | 1 | 1 | 0 | **YES** |
| **PD6** | 1 | 1 | 1 | 1 | 1 | 1 | **YES** | 1 | 1 | 1 | 1 | 1 | 1 | **YES** | 1 | 1 | 1 | 1 | 1 | 1 | **YES** |
| **PD7** | 1 | 1 | 1 | 1 | 0 | 0 | **YES** | 1 | 1 | 1 | 1 | 0 | 0 | **YES** | 1 | 1 | 1 | 1 | 0 | 0 | **YES** |
| **PD8** | 1 | 0 | 1 | 0 | 1 | 0 | **YES** | 1 | 0 | 1 | 0 | 1 | 1 | **YES** | 1 | 1 | 1 | 1 | 1 | 1 | **YES** |
| **PD9** | 1 | 1 | 1 | 1 | 1 | 1 | **YES** | 1 | 1 | 1 | 1 | 1 | 1 | **YES** | 1 | 0 | 1 | 0 | 1 | 0 | **YES** |
| **PD10** | 1 | 1 | 1 | 1 | 1 | 1 | **YES** | 1 | 1 | 1 | 1 | 1 | 1 | **YES** | 1 | 1 | 1 | 1 | 1 | 1 | **YES** |
| **PD11** | 1 | 1 | 1 | 0 | 1 | 0 | **YES** | 0 | 1 | 1 | 1 | 1 | 1 | **YES** | 1 | 1 | 0 | 1 | 1 | 1 | **YES** |
| **PD12** | 1 | 1 | 0 | 1 | 0 | 1 | **YES** | 1 | 1 | 0 | 1 | 1 | 1 | **YES** | 1 | 1 | 0 | 1 | 1 | 1 | **YES** |
| **DT1** | 1 | 0 | 1 | 0 | 1 | 0 | **YES** | 0 | 0 | 0 | 0 | 1 | 1 | **NO** | 0 | 0 | 1 | 0 | 1 | 1 | **NO** |
| **DT2** | 1 | 1 | 0 | 0 | 0 | 0 | **NO** | 1 | 0 | 1 | 0 | 0 | 0 | **YES** | 1 | 0 | 1 | 0 | 0 | 0 | **YES** |
| **DT3** | 1 | 1 | 0 | 0 | 0 | 0 | **NO** | 1 | 1 | 0 | 0 | 0 | 0 | **NO** | 0 | 1 | 0 | 0 | 0 | 0 | **NO** |
| **DT4** | 1 | 1 | 1 | 0 | 1 | 0 | **YES** | 0 | 0 | 1 | 0 | 0 | 0 | **NO** | 0 | 0 | 1 | 0 | 1 | 0 | **NO** |
| **DT5** | 1 | 1 | 1 | 0 | 1 | 0 | **YES** | 1 | 0 | 1 | 0 | 0 | 1 | **YES** | 1 | 0 | 0 | 0 | 0 | 1 | **NO** |
| **DT6** | 1 | 0 | 0 | 1 | 0 | 0 | **NO** | 0 | 0 | 0 | 0 | 0 | 0 | **NO** | 0 | 0 | 0 | 0 | 0 | 0 | **NO** |
| **DT7** | 1 | 0 | 1 | 0 | 1 | 0 | **YES** | 1 | 0 | 0 | 0 | 1 | 1 | **YES** | 0 | 0 | 0 | 0 | 1 | 1 | **NO** |
| **DT8** | 1 | 1 | 1 | 1 | 0 | 0 | **YES** | 0 | 0 | 0 | 0 | 0 | 0 | **NO** | 1 | 1 | 1 | 1 | 0 | 0 | **YES** |
| **DT9** | 1 | 1 | 1 | 1 | 0 | 1 | **YES** | 0 | 0 | 0 | 0 | 0 | 0 | **NO** | 0 | 1 | 1 | 0 | 0 | 0 | **NO** |
| **DT10** | 0 | 0 | 0 | 0 | 0 | 0 | **NO** | 0 | 0 | 1 | 0 | 0 | 1 | **NO** | 1 | 0 | 1 | 0 | 0 | 1 | **YES** |
| **DT11** | 1 | 1 | 0 | 1 | 1 | 1 | **YES** | 0 | 1 | 0 | 0 | 0 | 1 | **YES** | 0 | 1 | 0 | 1 | 0 | 1 | **YES** |
| **DT12** | 0 | 0 | 0 | 0 | 1 | 0 | **NO** | 0 | 0 | 0 | 1 | 0 | 1 | **NO** | 0 | 0 | 0 | 1 | 0 | 1 | **NO** |
| **HC1** | 0 | 0 | 0 | 0 | 0 | 0 | **NO** | 0 | 0 | 0 | 0 | 0 | 0 | **NO** | 0 | 0 | 0 | 0 | 0 | 0 | **NO** |
| **HC2** | 1 | 1 | 0 | 0 | 0 | 0 | **NO** | 0 | 0 | 0 | 0 | 0 | 1 | **NO** | 1 | 1 | 0 | 0 | 0 | 0 | **NO** |
| **HC3** | 1 | 1 | 1 | 0 | 1 | 0 | **YES** | 0 | 1 | 0 | 0 | 0 | 0 | **NO** | 0 | 1 | 0 | 1 | 0 | 1 | **YES** |
| **HC4** | 0 | 1 | 1 | 0 | 1 | 1 | **YES** | 0 | 1 | 1 | 0 | 1 | 1 | **YES** | 0 | 1 | 1 | 0 | 1 | 1 | **YES** |
| **HC5** | 0 | 1 | 1 | 0 | 0 | 0 | **NO** | 1 | 1 | 0 | 0 | 0 | 0 | **NO** | 0 | 1 | 0 | 0 | 0 | 0 | **NO** |
| **HC6** | 0 | 0 | 0 | 0 | 0 | 0 | **NO** | 0 | 0 | 0 | 0 | 0 | 0 | **NO** | 0 | 0 | 0 | 0 | 0 | 0 | **NO** |
| **HC7** | 0 | 0 | 0 | 0 | 0 | 0 | **NO** | 0 | 0 | 0 | 0 | 0 | 1 | **NO** | 0 | 0 | 0 | 0 | 0 | 1 | **NO** |
| **HC8** | 1 | 1 | 1 | 1 | 1 | 1 | **YES** | 1 | 1 | 1 | 1 | 0 | 0 | **YES** | 1 | 1 | 1 | 1 | 0 | 0 | **YES** |
| **HC9** | 1 | 0 | 1 | 0 | 1 | 0 | **YES** | 1 | 0 | 1 | 0 | 0 | 1 | **YES** | 1 | 0 | 0 | 0 | 0 | 1 | **NO** |
| **HC10** | 0 | 0 | 0 | 0 | 0 | 0 | **NO** | 0 | 0 | 0 | 0 | 0 | 0 | **NO** | 0 | 0 | 0 | 0 | 0 | 0 | **NO** |
| **HC11** | 1 | 1 | 0 | 0 | 0 | 0 | **NO** | 0 | 1 | 0 | 0 | 0 | 0 | **NO** | 1 | 1 | 0 | 1 | 0 | 0 | **NO** |
| **HC12** | 0 | 0 | 0 | 0 | 0 | 0 | **NO** | 0 | 0 | 0 | 0 | 0 | 0 | **NO** | 0 | 0 | 0 | 0 | 0 | 0 | **NO** |

**Description of polymyographic assessment of patients with dystonic tremor**

Recording electrodes were placed on biceps brachii, extensor carpi radialis, flexor carpi radialis and the first dorsal interosseal muscle, with accelerometer attached to the distal phalanx of the index finger of more severely affected limbs in the following situations:

- at rest - in basic position, after tactile, nociceptive and proprioceptive stimulation, during calculation 100-7 and during contralateral motor task
- in posture - extended wrist with supported forearm, extended wrist with supported elbow, extended elbow and wrist, extended wrist with a 1 kilogram weight
- in action - alternating hand flexion/extension in wrist, alternating forearm flexion/extension in elbow, finger - nose test, spiral drawing
- distraction techniques (ballistic movement of contralateral limb) were used in situations with the most prominent tremor.

During examination, we recorded the presence of:

- tremor - frequency, amplitude and modifying factors
- myoclonus - burst duration, temporal and spatial characteristics, provocation factors
- dystonic features - null point, mirror movements/dystonia, overflow of motor activity, antagonists co-contraction.
